# The Wnt and BMP Families of Signaling Morphogens at the Vertebrate Neuromuscular Junction

**DOI:** 10.3390/ijms12128924

**Published:** 2011-12-05

**Authors:** Juan P. Henríquez, Catherine E. Krull, Nelson Osses

**Affiliations:** 1 Laboratory of Developmental Neurobiology (LDNB), Department of Cell Biology, Faculty of Biological Sciences, University of Concepcion, and CMA Bio-Bio, Concepcion 4089100, Chile; 2 University of Michigan, 5211 Dental, Ann Arbor, Michigan, MI 48109, USA; E-Mail: krullc@umich.edu; 3 Institute of Chemistry, Faculty of Sciences, Pontificia Universidad Católica de Valparaíso, Valparaiso 2340025, Chile; E-Mail: nelson.osses@ucv.cl

**Keywords:** Wnt, BMP, neuromuscular junction

## Abstract

The neuromuscular junction has been extensively employed in order to identify crucial determinants of synaptogenesis. At the vertebrate neuromuscular synapse, extracellular matrix and signaling proteins play stimulatory and inhibitory roles on the assembly of functional synapses. Studies in invertebrate species have revealed crucial functions of early morphogens during the assembly and maturation of the neuromuscular junction. Here, we discuss growing evidence addressing the function of Wnt and Bone morphogenetic protein (BMP) signaling pathways at the vertebrate neuromuscular synapse. We focus on the emerging role of Wnt proteins as positive and negative regulators of postsynaptic differentiation. We also address the possible involvement of BMP pathways on motor neuron behavior for the assembly and/or regeneration of the neuromuscular junction.

## 1. Introduction

The vertebrate neuromuscular junction (NMJ) has been extensively used as a model of synaptogenesis essentially due to its larger size and simplicity, as compared to central synapses. As a consequence, several proteins that have been demonstrated to play crucial roles in the behavior of central synapses, were first described at the NMJ. In addition, the NMJ is the final target of therapeutic strategies aimed to regenerate peripheral synaptic connections after neurodegenerative or traumatic injury of the spinal cord. In this regard, it is fair to assume that the more we increase our knowledge on the strategies employed by development in the original wiring of the nervous system, the more we will be able to precisely imitate them for successful regeneration. Therefore, research directed to unveil the mechanisms accounting for NMJ assembly, maturation and maintenance contributes to the basic knowledge of synaptic behavior as well as to potential strategies for regeneration.

At the molecular level, it has been demonstrated that both nerves and muscles secrete molecules that regulate key aspects leading to the assembly of functional neuromuscular synapses [1,2]. For instance, signaling proteins, either soluble or components of the extracellular matrix, act at both sides of this peripheral synapse to exert positive and negative effects on the differentiation of pre and postsynaptic terminals (for reviews, see [2–5]).

Cumulative evidence obtained from different model organisms, either invertebrate or vertebrate, has demonstrated that signaling molecules that act as morphogens during early development, such as Wnts and BMPs, regulate crucial events later that lead to the formation of proper neuronal connections. Members of both signaling pathways have been involved in several features of neuronal behavior, such as differentiation, axonal guidance and synaptogenesis.

In this review, we will first summarize current views on the *in vivo* role of well described molecules playing crucial roles on the establishment of pre and postsynaptic terminals at the vertebrate NMJ. We will additionally focus on current data, mainly obtained in invertebrate systems, indicating that Wnts and BMPs potentially activate different signaling pathways to modulate, positively and negatively, the formation of the vertebrate NMJ.

## 2. The Neuromuscular Junction

During vertebrate embryonic development, motor nerve trunks penetrate peripheral regions where myotubes have been recently differentiated. Later on, motor neuron axons branch to innervate a variable number of skeletal muscle fibers in a discrete central region of the muscle named the *end-plate band*. Presynaptic differentiation is accompanied by morphological changes in motor terminals that contact the muscle fiber and begin to accumulate synaptic vesicles containing acetylcholine and other presynaptic components. In turn, postsynaptic differentiation is characterized by increased expression of several genes coding for postsynaptic proteins, including the acetylcholine receptors (AChRs), in the few myonuclei localized beneath the junction and the aggregation of these proteins in a small invaginated and folded fraction of the muscle membrane to shape the mature NMJ [2]. The precise apposition of pre- and postsynaptic terminals observed in the mature NMJ is thought to be driven by reciprocal signaling between motor neurons and their target muscle fibers during early synaptogenesis. In this chapter, we will summarize relevant *in vivo* studies that have underscored the potential physiological role of different signaling molecules acting as pro and anti-synaptogenic signals at both sides of the vertebrate NMJ.

### 2.1. Presynaptic Differentiation at the Vertebrate NMJ

Even though little is known about the identity and function of muscle-derived molecules regulating *in vivo* presynaptic differentiation, there are good examples of signaling molecules affecting motor neuron behavior at the NMJ. One example of such proteins are members of the ephrin-A family of bidirectional signaling molecules, which are differentially expressed by developing skeletal muscles along the anteroposterior axis [6]. Consistent with *in vitro* experiments showing that rostral and caudal motor neurons bear different sensitivity to ephrin-A5 on neurite outgrowth [6], transgenic mouse models engineered to selectively overexpress ephrin-A5 or to silence both ephrin-A2 and -A5 showed abnormal topographic innervation by motor neurons and defective NMJs. Therefore, the conclusions of these studies point to a key role of ephrin-A proteins on motor terminals to induce the formation of specifically positioned neuromuscular synapses [6].

A comprehensive genetic approach conducted by Fox and colleagues analyzed the potential role of members of the fibroblast growth factor (FGF) family and extracellular matrix proteins, such as laminins and collagens, in presynaptic differentiation at the NMJ [7]. Interestingly, targeted mutation of these proteins *in vivo* showed that they control different sequential features of the vertebrate NMJ formation. Thus, whereas signaling through the FGF receptor 2b is required for the onset of presynaptic terminals, the presence of laminin-β2 is crucial for their maturation [7]. Interestingly, laminin-β2 binds to voltage-gated calcium channels in the presynaptic membrane [8], which have been recently found to form a binding complex with α3-integrins, cytoskeletal elements and active zone components at the mature NMJ [9]. On the other hand, collagen IV is required to maintain proper NMJs [7]. Remarkably, these functional findings strongly correlate with the differential spatiotemporal expression patterns of these proteins *in vivo* [7,9]. Taken together, *in vivo* approaches reveal that multiple signaling pathways are likely required to refine the correct differentiation and positioning of functional presynaptic terminals at the vertebrate neuromuscular synapse.

### 2.2. Neural Control of Postsynaptic Differentiation at the Vertebrate NMJ

The idea that neural inputs induce postsynaptic differentiation is supported by genetic studies showing that ablation of specific genes expressed by motor neurons results in severe defects in the morphology of the NMJ [10–12]. In agreement with these findings, early AChR clustering has been traditionally believed to be modulated by diffusible neural-derived factors that induce the synthesis and aggregation of postsynaptic proteins at the vertebrate NMJ [2,4].

Agrin is a motor neuron-secreted heparan sulfate proteoglycan extensively characterized by its ability to aggregate AChRs and other postsynaptic proteins in cultured muscle cells [13–17]. In support of its key role during postsynaptic differentiation, mice lacking agrin display severe defects in NMJ morphology [10]. Diaphragms of agrin-deficient mice contain much less and smaller AChR clusters, distributed in an abnormally wider end-plate band [10,18]. At the muscle membrane, agrin activates the muscle-specific tyrosine kinase receptor MuSK, which is concentrated in postsynaptic densities [19–21]. Intracellularly, agrin signaling requires the synaptic protein rapsyn, which associates with high affinity to AChRs in postsynaptic muscle domains [22]. More recently, the cytoplasmic MuSK-binding proteins Dok-7 and Tid1 have been shown to be also crucial for postsynaptic differentiation at the vertebrate NMJ [23–25]. Indeed, mice deficient in MuSK, rapsyn, Dok-7 or Tid1 show no signs of postsynaptic differentiation; accordingly, cultured myotubes deficient in any of these proteins are unable to form AChR clusters in the presence of agrin [19,22,24,25]. Together, these data support an essential role for agrin/MuSK signaling to induce postsynaptic differentiation at the NMJ.

### 2.3. Aneural Signals Induce Postsynaptic Pre-Patterning at the Vertebrate NMJ

The crucial role of neural-derived molecules on postsynaptic differentiation has, however, been challenged by the findings showing that AChR clusters do form even before motor axons contact muscle fibers [18,26,27]. Furthermore, AChRs aggregate in an end-plate band during the earliest stages of NMJ development in agrin mutants [11]. Remarkably, skeletal muscles of animals lacking motor neurons also assemble a central profile of AChR clusters [11,28,29]. Even though the molecular mechanisms regulating aneural pre-patterning are poorly understood, impairment of MuSK function results in the complete loss of aneural AChR clusters [10,18], demonstrating that MuSK activation, possibly by ligands other than nerve-derived agrin, regulates pre-patterning. What is the function of nerve-independent AChR clusters? Studies in a mouse model engineered to assemble ectopic NMJs all along their muscle fibers suggest that aneural AChR clusters could determine the muscle membrane domains where neuromuscular synaptogenesis begins [30,31]. Consistently, in developing zebrafish some aneural AChR clusters are incorporated into nascent synapses [26,32]. However, rescue experiments after pre-patterning inhibition showed mislocalized, but still functional, mature neuromuscular synapses, revealing that aneural AChR clusters are not a requisite for NMJ assembly ([18], see below). Thus, whether mammalian NMJs incorporate pre-patterned postsynaptic specializations into developing synapses, and to what extent this is required for NMJ formation, is still to be determined.

### 2.4. The Neurotransmitter Acetylcholine Inhibits NMJ Assembly

A relevant emerging concept in the field is the existence of negative signals for postsynaptic differentiation that act at the most abundant non-innervated (“extrasynaptic”) domains of the muscle membrane. Several lines of evidence reveal that the neurotransmitter acetylcholine acts as such a signal. Indeed, mutant mice for choline acetyltransferase (ChAT), the sole synthetic enzyme for acetylcholine, display wider endplate bands containing more innervated AChR clusters than wild-type littermates [33,34]. In addition, ChAT/agrin double mutants rescued the inhibition of AChR clustering observed in agrin single mutants at late stages of NMJ assembly [33,34]. Interestingly, recent findings in animals rendered null only for muscle AChRs, reveal that the negative effects that ACh plays on AChR clustering seem not to rely on the activation of muscle receptors [35]. In addition, the “anti-synaptogenic” effect of acetylcholine is antagonized in synaptic domains by the “pro-synaptogenic” role of motor neuron-derived agrin [33–35]. As a general outcome of these genetic approaches, the co-existence of positive and negative inputs is essential to refine the proper apposition of the pre- and postsynaptic terminals observed in functional NMJs.

## 3. Wnt Signaling at the Neuromuscular Synapse

Wnt ligands comprise a family of 19 secreted signaling proteins with a wide range of developmental functions including cell fate decisions during early embryogenesis and specific cellular responses such as cell proliferation, differentiation and survival [36]. Interestingly, Wnt ligands have also emerged as key molecules regulating crucial steps of neurodevelopment, including neuronal fate and differentiation, axonal guidance, dendritic development and synaptogenesis (for reviews, see [37,38]). The prevalent idea is that Wnt factors, which act as morphogens during early development, also regulate later, positively and negatively, crucial events resulting in the formation of proper neuronal connections [37,38]. Within this section we will focus on the emerging role of Wnt pathways during vertebrate NMJ assembly, taking as a basis what has been better described at the invertebrate neuromuscular synapse.

### 3.1. Wnt Pathways

Wnt ligands signal through their cognate seven-pass transmembrane G-protein coupled Frizzled (Fz) receptors from which ten members have been described in vertebrates [39]. Three different Wnt pathways can be activated after Wnt binding to Fz receptors and the subsequent activation of the cytoplasmic protein Dishevelled (Dvl) [40,41]. In the so-called “Wnt canonical” pathway, the glycogen synthase kinase-3β (GSK-3β) is inhibited, resulting in the intracellular accumulation of β-catenin which translocates to the nucleus where, along with Tcf/Lef1 transcription factors, activates the expression of specific Wnt target genes [39,42,43]. Two “non-canonical” pathways are also triggered by Wnts. The “Wnt calcium” pathway regulates cell fate decisions and cell movement during development by increasing intracellular Ca^2+^ levels that activate specific protein kinases [44]. In turn, the “planar cell polarity” pathway involves modifications of the cytoskeleton through the small GTPases Rac and Rho and the *N*-terminal c-Jun kinase and functions mainly in polarity and morphogenesis [45].

Along with the three pathways described, Wnt proteins activate several other signaling cascades [46]. This heterogeneity is due in part to the existence of different membrane proteins, including receptors and regulatory proteins that act as co-receptors, including the LRP5/6 proteins, which are specifically required to activate canonical Wnt signaling [47–49]. Also, extracellular molecules, including the secreted Fz-related proteins (Sfrps), act as endogenous antagonists of Wnt signaling [50]. Therefore, the existence of a large number of Wnt ligands, receptors and modulatory proteins, either cytosolic or extracellular, and, as a consequence, the different signaling pathways they generate, illustrates the broad diversity of functions that Wnt proteins play in different cell types and developmental processes.

### 3.2. Wnts Play Pro- and Anti-Synaptogenic Roles at the Invertebrate NMJ

At the *Drosophila* NMJ, the Wnt orthologue *Wingless* (Wg) is secreted by motor terminals whereas its DFz2 receptor is expressed both at pre- and post-synaptic cells [51]. In agreement with this expression pattern, Wg affects pre- and postsynaptic behavior. Indeed, suppression of Wg at late larval stages results in the formation of aberrant synaptic boutons [51]. Ultrastructural analysis shows mislocalization of pre and postsynaptic terminals and defective active zones [51]. A novel mechanism accounts for Wg effects on postsynaptic differentiation, as a Wg/DFz2 complex is endocytosed and translocated to the periphery of the nucleus where a DFz2 *C*-terminal polypeptide is cleaved and transported into the nucleus to possibly activate transcription of target genes [52,53]. This pathway is crucial for NMJ structure, as blocking the internalization of the Wg/DFz2 complex in muscle cells results in severe defects in NMJ synaptic structure [52,53].

Wnt signaling also affects presynaptic differentiation at the *Drosophila* NMJ. In motor neurons, Wg signaling is transduced through GSK-3β to regulate the formation of synaptic boutons and the recruitment of synaptic components. Indeed, GSK-3β mutants display abnormal synaptic boutons and NMJ growth by affecting presynaptic differentiation through a mechanism involving local β-catenin-independent changes in the dynamics of the microtubule cytoskeleton [54,55].

In contrast to the positive effects of nerve-derived Wg on the development of *Drosophila* NMJs, studies in *C. elegans* have demonstrated that Wnt signaling inhibits neuromuscular synaptogenesis. In this model system, the most proximal segment of the DA9 motor neuron is normally asynaptic whereas the remaining fraction of the axon form functional synapses with muscles along the dorso-ventral axis [56]. Mutant worms for the Wnt/lin44 ligand and for the Fz/LIN-17 receptor display synaptic puncta in the asynaptic axonal region [56]. Wnt/lin44 is expressed by few cells located towards the nematode tail, whereas Fz/LIN17 expression is restricted to the axonal asynaptic segment. Interestingly, ectopic expression studies of Wnt/lin44 resulted in mislocalized expression of Fz/LIN17 in areas close to the Wnt/lin44 source [56]. These findings support a model where a posterior-anterior gradient of Wnt/lin44 positions its Fz/LIN17 receptor at specific areas along the axon to inhibit NMJ formation [56]. Taken together, data obtained in invertebrates suggest that Wnts exhibit pro- and antisynaptogenic activities to regulate the formation and/or distribution of the neuromuscular synapse.

### 3.3. Role of Wnt Ligands in Postsynaptic Differentiation of the Vertebrate NMJ

Important *in vivo* evidence supports a role for Wnt signaling during early steps of postsynaptic differentiation at the vertebrate NMJ. For instance, *Dvl1* mutant mice diaphragms display postsynaptic domains organized in a wider end-plate band than control littermates [57], a phenotype resembling that of mutants of motor neuron proteins such as agrin and ChAT [10,12]. In addition, they are consistent with previous *in vitro* data obtained in cultured muscle cells that positioned Dvl as a key organizer of postsynaptic differentiation by regulating the function of the agrin receptor MuSK [58]. Considering that Dvl is a common mediator of Wnt pathways, these results suggest that Wnt signaling could affect the assembly and function of the vertebrate NMJ.

Cell transplantation experiments in the developing chick wing showed that the exposure of muscle cells to the Wnt-binding inhibitor Sfrp1 decreases AChR clustering [57], revealing that endogenous Wnt ligands could be involved in early steps of postsynaptic differentiation. In this regard, evidence obtained at the developmental stages of mouse NMJ formation showed that Wnt3 is expressed by motor neurons of the lateral motor column [59]. Consistent with its potential role in postsynaptic assembly, cell transplantation of Wnt3-overexpressing cells in the chick wing increases the clustering of AChRs in developing skeletal muscles [57]. Experiments in cultured myotubes showed that Wnt3 acts together with agrin to induce postsynaptic differentiation [57]. It has been described that, depending on the cell context, Wnt3 has the ability to activate either the β-catenin pathway or non-canonical Rho GTPases-dependent signaling [60,61]. Experiments using the inhibitors Sfrp1, which precludes Wnt binding to Fz receptors, and Dickkopf-1, which specifically interacts with LRP5/6 and thus blocks canonical signaling, did not to affect the ability of Wnt3 to induce AChR clustering along with agrin [57]. In turn, these *in vitro* studies revealed that Wnt3 activates Rac1 in a more efficient way than agrin does, whereas agrin preferentially increases Rho activity, suggesting that Wnt and agrin signaling pathways cross-talk at the vertebrate NMJ (for a review, see [62]). Consistent with previous findings addressing a role for small GTPases on AChR clustering [63,64], Wnt3-mediated activation of Rac1 resulted in the formation of AChR microclusters, which are converted into full-size aggregates upon agrin-dependent activation of Rho [57]. Future research will help to elucidate the molecular mechanisms by which Wnt activates Rac1 in this context. Together, these studies point to a positive role of Wnt ligands in postsynaptic differentiation at the vertebrate NMJ via an anterograde mechanism involving the activation of a non-canonical, Rac1-dependent signaling pathway.

In sharp contrast with the effect of Wnt3, the highly identical ligand Wnt3a plays negative roles on AChR clustering [65]. In support of a physiological role at the NMJ, Wnt3a is expressed by embryonic mouse skeletal muscles during neuromuscular synapse assembly [65]. Pre-treatment of muscle cells with Wnt3a resulted in impaired AChR clustering; also, agrin-induced aggregates are dispersed by Wnt3a [65]. As an *in vivo* support of these findings, electroporation of Wnt3a in post-natal muscles dispersed stabilized AChR clusters of mature NMJs [65]. In cultured muscle cells, the dispersal activity of Wnt3a was shown to be mediated by a mechanism involving β-catenin-dependent, but TCF-independent, signaling that results in the down-regulation of rapsyn [65]. Consistently, mice null for β-catenin in skeletal muscles, but not in neurons, develop bigger AChR clusters distributed in wider end-plate bands than controls [66], thus supporting the notion that postsynaptic differentiation at the NMJ is negatively affected by a β-catenin-dependent pathway.

More recent evidence demonstrates that Wnt ligands also affect early aneural pre-patterning of AChR clusters at the vertebrate NMJ [18]. In zebrafish, down-regulation of the Wnt11r ligand induced strong defects in AChR pre-patterning and axonal branching, similar to those of mutants for the agrin receptor MuSK [67,68]. Indeed, Wnt11r interacts genetically and biochemically with the ligand-binding domain of MuSK [18]. These functional studies revealed that Wnt ligands, through MuSK, modulate the pre-patterning of AChR clusters and the guidance of motor axons.

**Figure 1:**
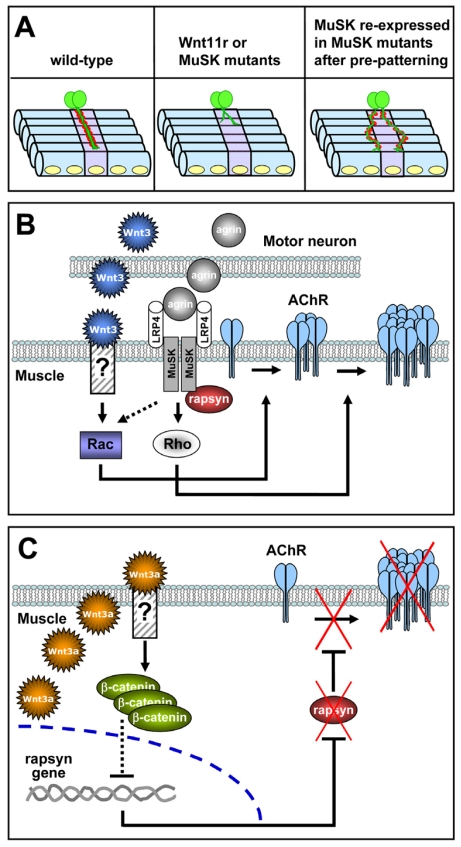
Wnt ligands modulate postsynaptic differentiation at the vertebrate NMJ. (**A**) Wnts induce aneural AChR clustering. In zebrafish, Wnt11r silencing results in strong axonal branching defects associated with impaired AChR pre-patterning, similar to MuSK null mutants (*middle panel*). MuSK rescue after pre-patterning inhibition (induced by MuSK depletion), resulted in mislocalized, but still functional, neuromuscular synapses (*right panel*); (**B**) Wnts as positive cues for postsynaptic differentiation. Wnt3 and agrin released from motoneurons collaborate to promote the formation of AChR clusters. Wnt3-induced AChR microclusters via Rac1 are converted into large clusters by agrin, which promotes the further activation of Rac1 and Rho; (**C**) Wnt-dependent disaggregation of AChR clusters. Wnt3a, secreted by muscle cells at the stages of NMJ formation, activates a β-catenin pathway that induces the dispersal of AChR clusters through the inhibition of rapsyn expression.

In summary, Wnt ligands affect different features of the vertebrate NMJ formation. Regarding postsynaptic differentiation, they have the ability to modulate the early assembly of aneural AChR clusters, possibly to distribute nascent neuromuscular synapses. In areas of synaptic contact, motor neuron-derived Wnts could act through local pathways as positive inputs for postsynaptic differentiation; in turn, Wnt/β-catenin signaling, possibly activated by non-neuronal Wnt ligands, could negatively regulate postsynaptic differentiation in non-innervated muscle regions [62] (Figure 1). Even though a role for Wnt signaling in presynaptic differentiation has been well documented in central neurons [69–71], as well as in the *Drosophila* NMJ [54,55], a potential role for Wnt pathways in the synaptic differentiation of vertebrate motor neurons still remains to be elucidated.

## 4. BMP Signaling Pathways at the Neuromuscular Synapse

Bone morphogenetic proteins (BMPs) belong to the transforming growth factor-β (TGF-β) superfamily and were originally named for their ability to induce ectopic bone formation [72,73]. Over 20 BMP family members have been identified, which can be classified in multiple subgroups based on sequence similarities of highly related proteins including homologues in *Drosophila* and *C. elegans* [74].

Besides their role in bone formation, different studies have shown that BMPs are multifunctional proteins with effects on diverse biological processes ranging from patterning and specification of several tissues and organs during embryonic development to adult tissues homeostasis. Genetic studies in *Xenopus*, *Drosophila*, zebrafish and mouse have shown the central role of BMPs during the development of dorsal-ventral patterning, patterning of the body axes, early patterning of the central nervous system, skeletogenesis, and development of heart, kidney, eyes and limb buds, among others [74–77].

In neuronal adult tissue, BMPs regulate several features of cell behavior. For instance, dendritogenesis, number of neurites, length of neurites and branch points have been shown to be stimulated or inhibited by different BMPs in diverse neuronal types, including cultured sympathetic, cortical, hippocampal, cerebellar and peripheral neurons [78–84]. The effects of different BMPs on morphological differentiation of neurons are likely to depend on the specific molecular determinants that mediate their signaling. In this chapter, we will summarize the well described role of BMP signaling at the *Drosophila* NMJ. Additionally, we will focus on some *in vivo* evidence obtained in vertebrate models, showing changes in the expression of BMP signaling effectors upon spinal cord injury and regeneration. We discuss these findings on the light of the potential role that BMP pathways may have during vertebrate NMJ assembly and regeneration.

### 4.1. BMP Signaling Pathways

Early events in BMP signaling involve the formation of heteromeric complexes of two types of transmembrane receptors with serine/threonine kinases activity, named type I and type II. BMPs binding to a preformed heteromeric complex of BMPRII and BMPRI initiate the classical Smad signaling pathway [85,86]. Upon BMP binding, BMPRI is phosphorylated by BMPRII. Activated BMPRI initiate the phosphorylation of specific receptor-regulated Smad proteins, namely R-Smad-1, -5 or -8. After phosphorylation, R-Smads form heteromeric complexes with the common mediator Smad-4. These Smad complexes migrate to the nucleus and regulate the transcription of specific target genes [87–89]. In addition, BMPs also have the ability to trigger Smad-independent pathways, which are mediated by different intracellular mediators, including p38 MAP kinase, ERK, nuclear factor kappa B (NFκB), and the phosphoinositide 3-kinase [76,90]. The wide range of different effects of BMPs, based on the mentioned signaling mechanisms, is extremely dependent on the cell context. This feature is due to the existence of several regulatory factors, such as antagonists that bind and inactivate the ligands, co-receptors present at the cell surface acting positively or negatively, by intracellular regulatory proteins, and by the expression of specific co-repressors or co-activators that regulate the transcription of specific target genes [76,87].

**Figure 2:**
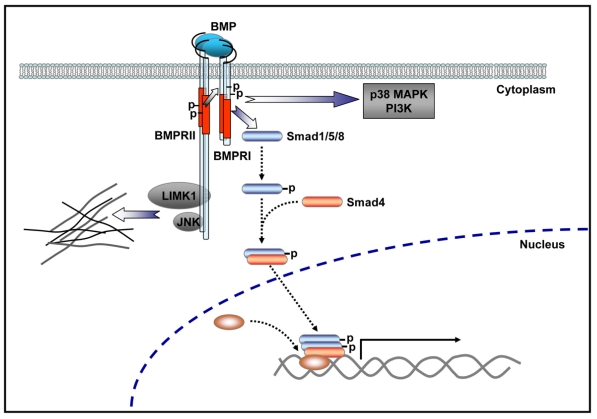
BMP signaling. Smad signaling is initiated upon phosphorylation of Smad-1, -5 or -8 by a heteromeric complex of BMPRII, BMPRI and BMP ligand. Phosphorylated Smads recruit Smad-4 and translocate to the nucleus where, in association with co-repressors or co-activators, regulate the transcription of specific target genes. Non Smad pathways, such as p38 MAP kinase and PI3 kinase, are also initiated by heteromeric complexes. The long cytoplasmic tail of BMPRII binds to cytoskeleton regulators mediating actin remodeling (LIMK1) and microtubule stabilization (JNK).

An interesting feature of BMP-mediated signaling is the fact that BMPRII bears a long *C*-terminal tail following its kinase domain that is not required to induce Smad dependent or independent signaling [86]. Remarkably, this long *C*-terminal tail of BMPRII is also present in its corresponding homologues in *C. elegans*, *Drosophila* and *Xenopus* [91–93]. Some of the reported BMPRII cytoplasmic tail interacting proteins correspond to the regulators of cytoskeleton dynamics LIM kinase 1 (LIMK1) [94], the c-Jun *N*-terminal kinase (JNK) [95], and the dynein light chain family member Tctex1 [96]. In a neuronal context, BMP-7-induced dendritogenesis is dependent on LIMK1-mediated actin remodeling and JNK-mediated microtubule stabilization. Both activities of cytoskeleton regulators rely on their binding to the BMPRII cytoplasmic tail [82,95]. Thus, BMP signaling through BMPRII activates key local pathways related to dendritogenesis and neurite outgrowth. The involvement of BMPRs in different signaling mechanisms is summarized in Figure 2.

### 4.2. BMP Signaling at the Invertebrate NMJ

Genetic and biochemical evidence obtained at the invertebrate NMJ shows that retrograde BMP signaling plays crucial roles in presynaptic motor neurons to regulate synaptic growth, assembly and maintenance [91,97–101]. Studies in *Drosophila* have demonstrated that a key component of BMP signaling affecting the NMJ is *wishful thinking* (*Wit*), the BMPRII homolog, as *Wit* mutant larvae show a significant reduction in size and function of the NMJ [91,99]. *Wit* mutants showed specific loss of the phosphorylated form of the Smad homolog Mad in embryonic motor neurons and decreased neurotransmitter release. In addition, ultrastructural examination of the neuromuscular synapse showed complete presynaptic membrane detaching from the postsynaptic membrane in certain areas [91,99]. Importantly, the *Wit* mutant phenotype could be largely rescued by the expression of Wit in motor neurons, revealing a major function of this receptor in presynaptic cells at the invertebrate neuromuscular synapse [91,99].

Since Wit function is required in the presynaptic cell, it could transduce signals from the muscle to the motor neuron cell to coordinate synaptic growth with muscle growth. Impaired motor neuron synaptic growth and function has also been demonstrated in loss of function mutants either for the BMP homolog Glass bottom boat (Gbb), the BMPRI Saxophone (Sax) and/or Thickveins (Tkv) or the Smad homologs Mad and Medea [98,102–104]. Hence, a retrograde signal activates canonical BMP signaling that works through a transcriptional mechanism in the cell soma to control motor neuron synaptic growth at the NMJ. In agreement, Gbb expression in muscle, but not in neurons, rescues NMJ size defects observed in Gbb mutants and disruption of retrograde axonal transport inhibits BMP signaling in motor neurons [102]. Moreover, Gbb and Mad mutants, as well as retrograde transport inhibition, diminished transcription of the Rac GEF *trio* in motor neurons, which is required for normal synaptic growth at the NMJ. Gbb expression in muscle and overexpression of activated Tkv and Sax in motor neurons enhance the transcription of *trio* [97]. Even though Gbb is also expressed in presynaptic neurons, recent evidence points to the muscle-derived Gbb pool as the main signal at the NMJ for synaptic growth [101]. Overall, these findings support a model where retrograde signals from the muscle activate Smad dependent pathways in motor neurons, leading to the expression of target genes that play crucial roles during synaptic growth and morphology.

**Figure 3:**
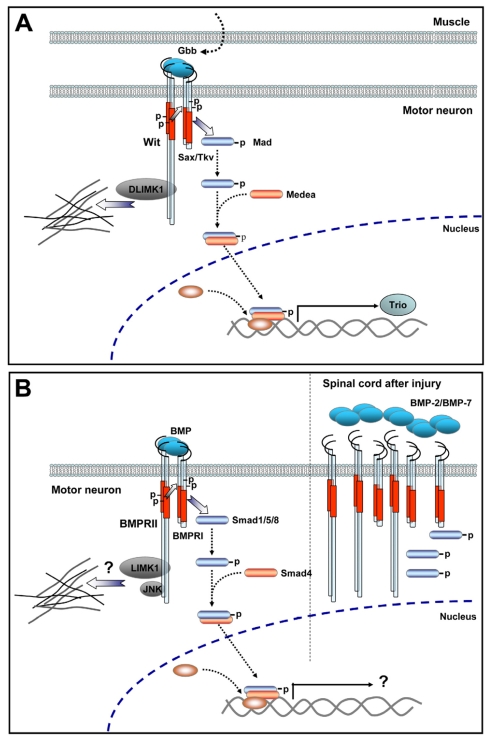
BMP signaling in motor neurons. (**A**) In *Drosophila*, the evidence suggests that a retrograde signal involving homologues of the BMP pathway regulates synaptic growth, assembly and maintenance by pathways related to either Smad or cytoskeleton regulators; (**B**) In vertebrates, spinal cord injury induces an increase of BMP ligands around the injury site. BMP receptors and Smad1 phosphorylation is also increased in spinal neurons, revealing the ability of BMP to transduce Smad-dependent signaling. However, the target genes induced by Smad pathways or the mechanisms involving local cytoskeletal rearrangement are unknown.

In addition, mutants of the canonical BMP signaling cascade, including Gbb, Wit, Tkv, Mad and Medea, showed an increased number of sites of “synaptic footprints”, organized postsynaptic muscle membranes that lack opposing presynaptic neuronal markers; therefore, synaptic footprints identify regions of the NMJ where the terminal nerve once resided and has retracted [98]. These findings involve, in addition to synaptic growth, a potential role of BMP signaling in synaptic stability. In this regard, mutants of Wit receptor showed a strong increase of synaptic footprints as compared to those observed in mutants of other canonical BMP signaling molecules. A potential mechanism accounting for this effect is related to the ability of the *C*-terminal domain of *Wit* to interact with the actin cytoskeleton modulator DLIMK1 in motor neurons to stabilize the NMJ. Remarkably, this interaction is not required for Smad-mediated synaptic growth [98]. Therefore, Smad-dependent and independent signaling pathways via the Wit receptor in invertebrate motor neurons are involved in synaptic growth and stability at the NMJ (Figure 3A).

Interestingly, *Drosophila* models of motor diseases have also been connected to BMP signaling. Hereditary Spastic Paraplegia (HSP) is a group of genetic disorders characterized by retrograde axonal degeneration of the corticospinal tract and the posterior columns in the spinal cord that result in progressive spasticity and weakness of the lower limbs [105]. Mutants of Spichthyin (Spict), the *Drosophila* ortholog of the human HSP gene NIPA1 (Nonimprinted in Prader-Willi/Angelman), display a 2-fold increase in synaptic boutons. Importantly, presynaptic overgrowth at the NMJ in mutants is recovered by the specific expression of Spict in neurons, as well as by mutations in Tkv, Sax, Wit, Gbb and Medea, suggesting that Spict inhibits the BMP pathway in motor neurons [106]. It is interesting to point out that whereas some motor diseases, such as HSP, show a relationship with increased BMP signaling, other *Drosophila* models of motor diseases, such as amyotrophic lateral scelerosis and spinal muscular atrophy, correlate with down-regulation of BMP signaling (for reviews, see [107,108]). At any rate, evidence from invertebrate models reveals a crucial role for BMP signaling in the physiological and pathological behavior of motor neurons.

### 4.3. In Vivo Evidence Supports a Role for BMP Signaling in Vertebrate Motor Neurons

A physiological role for BMPs in adult motor neurons comes from experimental models of injury. For instance, the expression of BMP-2 mRNA is induced in motor neurons after crush injury of the facial nerve in rabbits [106]. Similarly, traumatic injury of the rat spinal cord resulted in a remarkable up-regulation of BMP-7 and BMP-2 around the injury site during recovery [109,110]. In functional experiments, enhanced locomotor activity and axonal regrowth have been observed when BMP binding to their receptors is inhibited by administration of noggin —a high affinity soluble antagonist of BMP-2— into the injured spinal cord [109]. Accordingly, experiments where adult mouse spinal cords were injured by compression showed a partial functional recovery after transplantation of neural progenitor cells modified to express noggin [111]. In sharp contrast with these findings, transplantation of neural stem cells virally transfected to secrete noggin into rat spinal cord injured either by contusion or ischemia resulted in detrimental effects, including increased lesion volumes [112]. Although the discrepancies for these results remain to be elucidated, one possible explanation is that functional recovery is achieved when noggin or noggin-expressing cells are delivered at the time of injury [109,111] and not at later stages [112]. Indeed, it has been shown that BMP-2, BMP-4 and BMP-7 expression levels began to decrease four days post injury and no significant differences are detected later between injured and normal uninjured spinal cords [113].

Spinal cord regeneration is strongly impaired by the formation of a glial scar that inhibits axonal regrowth at injured sites [114]. Since BMPs induce astroglial differentiation, manipulation of BMP signaling at the injured spinal cord has been mainly intended to avoid the restriction of axonal regrowth and, therefore, to accomplish functional recovery [115]. Nevertheless, little is known regarding the physiological role of BMP signaling on adult motor neuron cells. In spite of the data showing that the recovery of contusion-induced spinal cord injury is accompanied by up-regulation of BMPRII, BMPRIA and Smad phosphorylation in spinal neurons [109], Smad1 mRNA and protein levels are up-regulated in motor neurons after hypoglossal nerve injury in rats [116]. In addition, BMP-2, -4 and -7 are expressed in ChAT-positive motor neurons in the gray matter of injured adult spinal cords [113]. When cultures of neural stem cells derived from adult spinal cord were treated with BMP-4, neurons display fewer and shorter processes, suggesting that BMP signaling delays neuronal maturation [113]. It is interesting to hypothesize that BMP signaling allows the synthesis of relevant proteins for axon growth or for synapse assembly, thus preventing precocious differentiation. Accordingly, recent evidence has shown that direct activation of BMP/Smad1 pathway in adult dorsal root ganglion neurons results in induction of regeneration markers and restoration of axon growth potential in sensory neurons in a mouse model of spinal injury [117]. Together, these findings suggest a direct effect of BMP signaling in vertebrate motor fibers during the axonal regeneration of peripheral nerves after injury (Figure 3B).

### 4.4. BMP Signaling Modulation by Proteins Involved in Vertebrate Motor Neuron Dysfunction

Different mutated genes have been identified in HSPs [118]. Atlastin, spastin, spartin and NIPA1 are among the mutated HSP proteins accounting for the majority of clinical cases. Remarkably, common features of these proteins in vertebrates are their localization in endosomal membrane traffic compartments and their ability to influence BMP signaling [119,120].

For instance, morpholino-dependent knockdown of the ortholog of human atlastin (*atl1*) in developing zebrafish induced a prominent loss of larvae motility, associated with abnormal axon pathfinding and multiple aberrant branching of spinal motor neurons [120]. Remarkably, primary cultures of zebrafish spinal neurons from *atl1* morphants showed a significant increase in the amount of nuclear pSmad1/5/8, suggesting that atlastin inhibits BMP signaling. Consistently, *atl1* knockdown defects were rescued by genetically or pharmacologically inhibiting the BMP pathway [120]. As for atlastin, enhanced expression of pSmad1/5 has been observed after siRNA-mediated silencing of NIPA1, spastin and spartin in mammalian cells [119]. Together, these data support that different endosomal proteins related to HSP act as inhibitors of BMP signaling.

Even though little is known regarding the molecular mechanisms by which HSP proteins modulate BMP signaling, recent evidence reveals that they could modulate the bioavailability of BMP receptors at the cell membrane. For instance, atlastin1 localizes to punctate vesicular structures in neurites and the cell soma of zebrafish spinal neurons, where it displays partial colocalization with BMPRI in late endosomes, which are distributed all along spinal axons [120]. Therefore, atlastin could modulate BMP signaling by regulating the trafficking of BMP receptors. On the other hand, NIPA1 in addition to inhibiting BMP signaling, interacts with BMPRII and promotes its trafficking from the plasma membrane to the lysosomal compartment [119]. Hence, these findings suggest that axonal degeneration related to endosomal HSP proteins are mediated by up-regulation of BMP signaling by a mechanism involving the traffic of BMPRs. In addition, they highlight the importance of BMP signaling on the behavior of vertebrate motor neurons. Interestingly, *Drosophila* mutants of Spict and zebrafish knockdown of *atl1* display motor neuron presynaptic overgrowth and abnormal morphological motor neurons, respectively [106,120]. These findings raise the possibility that overactivation of the BMP pathway in motor neurons has detrimental effects on the pathfinding of axons towards target cells as well as on the recognition of their proper synaptic partner. It is tempting to speculate that a similar condition could be occurring in motor nerves after spinal cord injury (Figure 3B).

## 5. Conclusions

In the present review we have focused on recent evidence revealing that the well described roles of Wnt and BMP signaling at the invertebrate neuromuscular synapse could also take place at the vertebrate NMJ. As in invertebrate species, *in vivo* evidence points to an anterograde effect of Wnt signals on postsynaptic differentiation, whereas BMP ligands act more likely through retrogradely-induced mechanisms to induce presynaptic effects. It is still to be determined if, as in *Drosophila*, Wnt signaling affects motor neuron behavior in the context of the vertebrate NMJ.

Remarkably, animal models of injured spinal cord show increased expression of BMP signaling molecules. In addition, BMP pathways are modulated by proteins involved in vertebrate motor neuron dysfunction. Therefore, even though the role of BMP signaling at the vertebrate NMJ has not been elucidated, this evidence points to a key role for BMPR-mediated signaling during vertebrate motor axon extension towards muscle cells for the assembly, maintenance and/or regeneration of the neuromuscular synapse. In this regard, the reported cross-talk between Wnt and BMP pathways [121,122] could provide fine-tuning mechanisms to modulate the formation, growth and/or stability of the vertebrate NMJ. Future studies will be required to address the possibility that both pathways work together in this context and will also contribute to clarify the potential use of Wnt and BMP signaling molecules as potential targets for the regeneration of motor axons after injury of peripheral nerves.
